# Are there really any predictive factors for a successful weight loss after bariatric surgery?

**DOI:** 10.1186/s12902-020-0499-4

**Published:** 2020-02-05

**Authors:** Diego Cadena-Obando, Claudia Ramírez-Rentería, Aldo Ferreira-Hermosillo, Alejandra Albarrán-Sanchez, Ernesto Sosa-Eroza, Mario Molina-Ayala, Etual Espinosa-Cárdenas

**Affiliations:** 1 Endocrinology department, Hospital de Especialidades. Centro Médico Nacional Siglo XXI, Instituto Mexicano del Seguro Social, Av. Cuauhtémoc 330, Col Doctores, Cuauhtémoc, 06720 Mexico City, CP Mexico; 2 UIM Enfermedades Endocrinas. Hospital de Especialidades UMAE Centro Médico Nacional Siglo XXI, Instituto Mexicano del Seguro Social, Mexico City, Mexico; 3 Experimental Endocrinology Research Unit. Hospital de Especialidades UMAE Centro Médico Nacional Siglo XXI, Instituto Mexicano del Seguro Social, Mexico City, Mexico; 4 Internal Medicine department. Hospital de Especialidades. Centro Médico Nacional Siglo XXI, Instituto Mexicano del Seguro Social, Mexico City, Mexico

**Keywords:** Bariatric surgery, Factors associated, Weight loss, Y-roux gastric bypass, One anastomosis gastric bypass

## Abstract

**Background:**

Currently, bariatric surgery is the most effective treatment for severe obesity and its metabolic complications; however, 15–35% of the patients that undergo bariatric surgery do not reach their goal for weight loss. The aim of this study was to determine the proportion of patients that didn’t reach the goal of an excess weight loss of 50% or more during the first 12 months and determine the factors associated to this failure.

**Methods:**

We obtained the demographic, anthropometric and biochemical information from 130 patients with severe obesity who underwent bariatric surgery in our institution between 2012 and 2017. We used self-reports of physical activity, caloric intake and diet composition. An unsuccessful weight loss was considered when the patient lost < 50% or more of the excess weight 12 months after surgery. We compared the characteristics between the successful and unsuccessful groups in order to find the factors associated with success.

**Results:**

We included 130 patients (mean age 48 ± 9 years, 81.5% were women). One year after surgery, 26 (20%) had loss < 50% EBW. Unsuccessful surgery was associated with an older age, previous history of hypertension, abdominal surgery or depression/anxiety, also the number of comorbidities and unemployment affected the results. These patients loss enough weight to improve some of their comorbidities, but they are more prone to regain weight 2 years after surgery.

**Conclusions:**

A fifth of the patients undergoing bariatric surgery may not lose enough weight to be considered successful by current standards. Some patients may benefit from the surgery in the short term, but they are more likely to regain weight after 2 years. The factors influencing this result are still controversial but may be population-specific. Early detection of the patients that are more likely to fail is imperative to establish additional therapeutic strategies, without denying them the opportunity of surgery or waiting for weight re-gain to occur.

## Background

Obesity is an important health problem worldwide. In Mexico, the last National Health and Nutrition Survey (ENSANUT 2016) [1], reported that the prevalence of overweight was 39.2% while obesity was 33.3%, which means that less than one third of the population is currently in a healthy weight. Approximately 2.9% of the population is categorized as having severe obesity with a Body Mass Index (BMI) of 40 kg/m^2^ or more.

Obesity is associated with comorbidities that increase morbidity and mortality among the affected population. It has been reported that for every 5 kg/m^2^ of BMI above the upper limit of normal of 25 kg/m^2^, the overall mortality is increased by 30% (HR 1.29 [95% CI 1.27–1.32]) [2]. A BMI of 30–35 kg/m^2^ reduces the life expectancy by 2 to 4 years and a BMI of 40–45 reduces it by 8 to 10 years [2].

So far, in patients with severe obesity or those with BMI > 35 kg/m^2^ and other comorbidities, bariatric surgery is the most effective therapeutic option to achieve long-term significant weight loss and to improve control or even achieve remission of the associated comorbidities [3]; however, it has also been reported that 15–35% of the patients that undergo bariatric surgery do not reach their goal for weight loss (defined as losing at least 50% of the excess weight) during the first 2 years after the procedure [3–6].

The factors traditionally associated with unsuccessful weight loss are behavioral problems, social and demographic factors, the surgical technique and even genetic polymorphisms. Age is one of the most consistent predictor factors, with older patients having worse results [7–9]. Patients with higher initial BMIs are also less likely to reach normal weights and a BMI > 50 kg/m^2^ has been reported as a risk factor for failure after bariatric surgery [7, 8, 10–12]. Regarding behavioral and psychosocial factors, Sheets and collaborators found that eating patterns such as binging and grazing, as well as depression are the most frequently associated with poor outcomes. On the other hand, adherence to the nutritional and exercise plans after surgery has been considered good prognostic factors for significant weight loss [13].

The factors associated with better prognosis remain controversial and may be particular to each population or multidisciplinary therapeutic approach. Since surgery is advocated for many patients with severe obesity, more information is needed to select the best candidates for surgery and to establish realistic goals for the intervention in each case. In some cases, the proficiency of the surgical procedure will not be enough to reach the desired weight loss goal. This, so called, failure calls into question the use of bariatric surgery in some cases. While the indications for surgery and follow up are somewhat clear, the indications for early interventions after surgery, for patients that are not losing enough weight, are not. Analyzing the reasons why this is happening, may contribute to create specific guidelines for these patients.

We aimed to determine the proportion of patients that didn’t reach the goal of an excess weight loss of 50% or more during the first 12 months in our center and to determine if there is any baseline clinical or laboratory characteristic that may be used as early predictors of this result.

## Methods

We performed a retrospective analysis of the information contained in the medical files of patients that requested an evaluation for bariatric surgery in our hospital between 2012 and 2017 and described their general characteristics. Our clinic accepts for this procedure only patients older than 18 years old, with a BMI > 40 kg/m^2^ or BMI > 35 kg/m^2^ plus one or more comorbidities, a documented failure to lose weight with diet and physical activity and approval by the psychiatry committee. For this study we further analyzed only the data of the patients that underwent the procedure and that were evaluated for a period of at least 1 year after the surgery. For this part of the analysis, we also excluded the files with incomplete information.

The study was authorized by the local ethics committee and followed the international Good Clinical Practice recommendations for the evaluation of clinical files and privacy regulations. The patients were fully informed of the aims of the study and were asked to sign an informed consent before the data collection.

We registered their general demographic and anthropometric data of each patient. Their medical history and general laboratory workup regarding comorbidities associated with obesity were also evaluated.

Patients are required to lose at least 5% of the excess body weight (%EBW) before surgery with the help of 3 evaluations by the nutritionist and at least 150 min/week of aerobic exercise, specially directed to their physical capabilities. The instructions are provided by the same trained nutritionists and physicians. This helps to control their comorbidities and demonstrates their ability to comply with a long term lifestyle change.

Physical activity is obtained from self-reports delivered by the patients, recording the total minutes per week of aerobic exercise; the calorie intake and diet composition is obtained from a 24 h registry before each evaluation with the nutritionist. A successful %EBW with bariatric surgery was considered when the patient lost 50% or more of the excess weight 12 months after surgery.

We present the data with median and standard deviations and medians with interquartilic ranges according to the variable distribution. Qualitative data is presented as frequencies and proportions. Bivariate analysis was performed using x^2^ and exact Fisher’s tests for qualitative variables and a Student’s T-test or Mann Whithney’s U for qualitative data. Logistic regression was performed including the variables that resulted significant from the bivariate analysis. A *p* < 0.05 was considered to be significant.

## Results

From a total of 368 patients that were evaluated in the obesity clinic during the data collection period, we included only the 130 that fulfilled the inclusion criteria.

The average age of the patients was 48 ± 9 years, 106 (81.5%) were women, 52% had a level of education of 9 years or more (equivalent to high school), 67% were married or living with a partner, 35% did not have full time job. (Table 1).

**Table 1:** Basal demographic characteristics, comorbidities and type of bariatric surgery of studied population (*n* = 130)

Age (years)	48 ± 9
Female	106 (81.5%)
Body weight at first evaluation (kg)	123.5 (109–133)
BMI at first evaluation (kg/m^2^) BMI > 50 kg/m^2^	48 (42–51) 52 (40%)
Loss of 10% or more EBW before surgery	46 (35%)
Impaired Fasting glucose Type 2 diabetes mellitus	41 (31%) 34 (26%)
Hypertension	74 (57%)
Dyslipidemia	38 (29%)
Hypothyroidism	36 (28%)
Previous cardiovascular event	6 (5%)
Sleep apnea	35 (27%)
Osteoarthrosis	27 (21%)
Previous abdominal surgery	66 (51%)
Depression or anxiety	18 (14%)
Type of surgery	
RYGB	49 (38%)
OAGB	64 (49%)
Gastric sleeve	17 (13%)

*BMI* body mass index, *EBW* excess body weight, *RYGB* Y-Roux Gastric Bypass, *OAGB* One anastomosis gastric bypass

During the initial evaluation at our clinic, 31% had elevated fasting plasma glucose and 26% were diagnosed with diabetes mellitus. Hypertension was present in 57% of them, dislipidemia in 29%, a previous cardiovascular event in 5% and history of deep venous thrombosis in 2%. Sleep apnea had been diagnosed in 27% while 21% had been treated for osteoarthrosis at some point. A history of depression or anxiety diagnosed by a mental health specialist was present in 14% of the patients.

The median BMI at first evaluation was 48(IQR 44–52) kg/m^2^. A shocking 40% of our population had a BMI of 50 kg/m2 or more, with 36.1% of them being considered super obese and 3.9% super-super obese.

The EBW% loss with diet and exercise interventions before surgery was 7% (IQR 0–12%), which resulted in a median weight at the time of surgery of 119 (104–128.5 kg) and a median BMI 46 (IQR 42–51) kg/m^2^. Only 35% of the patients lost more than 10% of the excess weight before the surgery.

Only two surgeons perform all the procedures in our center. They select the type of surgery according to their individual expertise and evaluation of the patients’ characteristics. The types of surgery available at our center are: Roux-en-Y bypass (RYGB), performed in 38% of the patients, laparoscopic one anastomosis gastric bypass (OAGB) performed in 49%, and gastric sleeve for the remaining 13%.

One year after surgery, 104 (80%) had loss 50% or more of the EBW. The patients with a successful surgery (Table 2), were younger than the patients in non-successful surgery group (46 ± 9, vs 52 ± 8 *p* = 0.005), had a lower frequency of hypertension (52% vs 77%, *p* = 0.02), abdominal surgeries (46% vs 69%, *p* = 0.03), depression or anxiety (11% vs 27%, p = 0.03). They also had a lower number of obesity-related comorbidities (3 vs 2, p = 0.02) and a higher proportion of patients lacking a full time job (53% vs 33%, *p* = 0.05).

**Table 2:** Comparison between the successful (> 50% EBW loss) vs non-successful surgeries (*n* = 130)

	Non-successful *n* = 26	Successful *n* = 104	p
Age (years) > 50 years of age at the time of surgery	52 ± 8 16 (61.5%)	46 ± 9 40 (38.5%)	0.005 0.04
Female	21 (81%)	85 (80%)	0.55 Fisher
Highschool or higher level of education.	12 (46%)	55 (53%)	0.53
Without a full time job	13 (54%)	33 (33%)	0.05
Married or living with a partner	20 (77%)	67 (64%)	0.22
Medical history			
Altered glucose metabolism	18 (69%)	57 (55%)	0.18
Hypertension	20 (77%)	54(52%)	0.02
Dyslipidemia	10 (38.5%)	28 (27%)	0.24
Hypothyroidism	9 (35%)	27 (26%)	0.37
Cardiovascular event	1 (4%)	5 (5%)	0.65
Venous thrombosis	0 (0%)	3 (3%)	0.50
Sleep Apnea	7 (27%)	28 (27%)	1.0
Osteoarthrosis	8 (31%)	19 (18%)	0.16
Previous abdominal surgery	18 (69%)	48 (46%)	0.03
Depression or anxiety	7 (27%)	11 (11%)	0.03
Total comorbidities	3 (3–4)	2 (1–3)	0.02
Before Surgery			
Maximum weight ever (kg)	131.5 (121–146.5)	130 (117–140)	0.39
Weight at initial evaluation (kg)	124.5 (113.5–135)	119.5(108–133)	0.319
BMI at first evaluation (kg/m^2^)	49 (46–53)	48 (43–53)	0.22
%EBW loss before surgery	7 (1.5–13)	3 (0–12)	0.29
Type of surgery:			
RYGB	13 (50%)	36 (35%)	0.001
OAGB	5 (19%)	59 (57%)	
Gastric sleeve	8 (31%)	9 (9%)	
EXERCISE AND DIET AFTER SURGERY			
At least 150 min of physical activity per week (%)	7 (27%)	32 (31%)	0.70
Average daily calorie intake during the 1st year (kcal)	846 (567.5–930)	723 (602–955)	0.94
Protein content of the diet (g)	50.5 (35–52)	47 (32–62)	0.82
Lipid content of the diet (g)	30 (16–39)	23 (17.5–33)	0.58
Carbohydrate content of the diet (g)	92.5 (58–109)	92 (73–128)	0.25
%EBW loss 12 months after surgery	43 (38–47)	66 (60–76)	< 0.001

*BMI* body mass index, *EBW* excess body weight, *RYGB* Y-Roux Gastric Bypass, *OAGB* One anastomosis gastric bypass

The surgery most likely to end in a successful outcome at this time point was OAGB, while the less successful in our group was the gastric sleeve (57% vs 9%, *p* = 0.001).

We found a significant inverse correlation between the baseline BMI and the %EBW lost at 12 months (R = − 0.356, *p* < 0.001), meaning that the patients with Grade II obesity were likely to lose most of the EBW (median of 91% of %EBW lost, IQR 85–98%), patients in grade 3 obesity had a %EBWL of 63.7% (IQR 52–72%), patients with super obesity lost 63.5% EBW (IQR of 54–66%) while the super super obese patients lost between only 57% of EBW (IQR 36–65%) during the first year. These percentages are deceitful, since the absolut kilogram loss was similar between groups (grade II obesity patients lost on average 46 kg during the first year, while the patients with super super obesity lost a mean of 54 kg, *p* = 0.076). Further analysis of these strata showed that the patients were also similar in terms of the presence of comorbidities and overall characteristics, the only difference was that the super obese and super super obese patients would probably require more time to reach the expected weight loss. If the average weight reduction for our group of patients was 50 kg/per year and the average EBW was 65.5 kg (IQR 55–79) and the EBW for patients with super super obesity was 93 kg (87–135), some of these patients will require more time to lose 50% of the EBW and will take 2 to 3 years to reach a normal BMI if they continue to lose weight at this rate, which is unlikely.

When we compared the patients with a successful versus non successful surgery at 12 months, there was no significant difference between their initial BMIs, the % EBW loss before surgery, the amount of exercise or calories registered or the content of the diet after surgery (carbohydrates, lipids and proteins).

We performed a multivariate analysis including age, hypertension, presence of a previous abdominal surgery, personal history of anxiety/depression disorder, lack of a full time job. Only the type of surgery‚ the lack of a full time job and a previous abdominal surgery persisted as negative predictors for a successful bariatric surgery. (Table 3).

**Table 3:** Multivariate analysis. Logistic regression of the variables associated to a successful weight loss after surgery (> 50% EBW)

Variable	Adjusted OR	P	IC 95%
Age	0.95	0.17	0.89–1.02
Not a full time job	0.26	0.04	0.07–0.96
Personal history			
Hypertension	0.44	0.18	0.13–1.46
Previous abdominal surgery	0.26	0.02	0.08–0.87
Depression / anxiety	0.26	0.05	0.07–1.01
Type of surgery	0.94	0.88	0.46–1.9

**Fig. 1:**
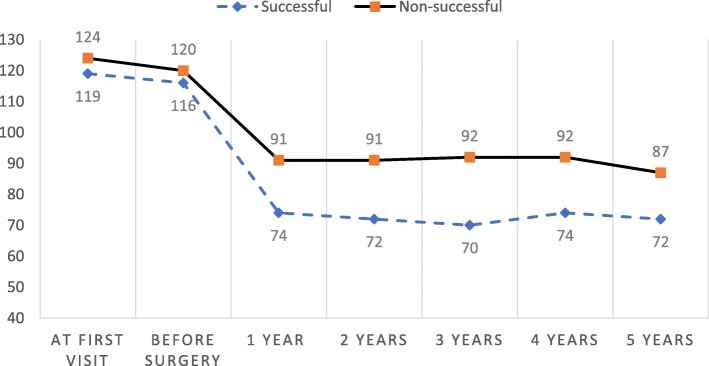
Graph 1. Weight variation (kg) before and after surgery of successful and non-successful groups

Since we also aimed to describe the particularities of the unsuccessful surgery group, we retrieved the long-term data available for 26 patients 2 years after surgery, 17 patients at 3 years, 10 patients at 4 years, and 7 patients at 5 years. In sum, 11 patients (42%) gained weight again when compared to the nadir reached after the first year from surgery, 3 (11%) remained stable and 12 (46%) managed to lose more weight after the first year of surgery; however, only 8 (30%) ultimately reached the goal of losing > 50% of the EBW at the end of their follow-up (delayed success) (Fig. 1). When comparing the characteristics of the patients with delayed success and those who never achieved the goals at any time during the follow-up, only the presence of alterations in glucose metabolism and lower % EBWL after the first year of surgery, were significantly different between the groups. The patients with delayed success had a lower prevalence of impaired glucose metabolism (55% in the delayed success vs. 100% in the never successful group, *p* = 0.03) and a higher % EBWL during the first year of surgery (47% vs 44% respectively, *p* = 0.01).

## Discussion

Bariatric surgery is currently considered to be the most effective therapy for weight loss and obesity-related comorbidities in patients with severe obesity; however, the definition of success is still under debate but it is clear that by using any proposed criteria, we will always find at least a few patients that will struggle to get to the goal weight and some of them will regain some of the weight after a few months. The risk-benefit balance for the successful surgeries is clearly in favor of performing the surgery in most cases, since not only the weight improves but also the number and severity of the obesity-related comorbidities is reduced. However, little attention is paid to the patients that do not reach the weight goals during the first months after the intervention. Other than considering them “surgery failures”, there are not clear guidelines as to what to do with these patients. Issues that need to be addressed include: the time and requirements for long term follow up visits, indications for additional supportive therapies and the possibility of establishing different long-term goals for different populations. It has been established that some patients fair better than others with these procedures using a standard criteria, maybe it is time to set a different criteria for special populations. For example, series evaluating the results for elderly patients that have undergone bariatric surgery acknowledge the fact that these patients will not lose as much weight as younger patients, but the improvement in their comorbidities, a goal that may not be reached even with polypharmacy (a major concern for gerontologists), may be reason enough to prescribe a surgery in some patients. A significant number of studies have shown that bariatric surgery improves some metabolic comorbidities soon after the surgery, results that cannot be attributed to the weight loss alone, but to the beneficial changes of the procedure in the neuroendocrine – gut axis, leading to the selection of some procedures over others when the metabolic benefits are needed even when patients are not severely obese. In our study, we found that the patients are mostly young female patients, which need to be reintegrated quickly to the workforce or to their family activities. The patients are seriously limited by their weight and they would represent a serious economic burden if they did not undergo surgery due to their long life expectancy but with a low quality of life. Super obese and super super obese patients may never reach the goal of a normal weight, but an improvement on their weight, a change of classification in the obesity stages may also reduce their complications and increase their functionality. However, these asseverations also need to be ascertained in the long term.

Many of the pitfalls in the long term management of severely obese patients, come from the lack of uniform criteria to determine success vs failure in bariatric surgery, but also because bariatric surgery is still struggling to become a mainstay therapy for obesity in many centers, leading to the fact that many physicians consider that the procedure must be proven effective for almost every patient before the experts can deal with the fact that not all patients may be the best candidates for it, or that surgery will be only the first of many surgical and medical interventions for these patients. We should also consider that realistic goals may be different for different patients and that even after the surgery was selected properly and executed flawlessly, some patients will still require additional long-term multidisciplinary treatments in order to reach and maintain the proposed goals. Just as with any other weight-loss strategy proposed so far, an efficacy of 100% with negligible adverse effects may not be possible, but more realistic goals to may help both the patient and the medical staff to decide if surgery is their best option and if so, which surgery is the most adequate. In this study we found that 1 out of every 5 patients that underwent bariatric surgery did not achieve the goal of losing 50% of the excess weight during the first 12 months of surgery, which is similar to the data reported by other authors [3]. This translates to the fact that 20% of all the patients that fulfilled the selection criteria will end up being considered to be a failed surgery and nothing more. Expert multidisciplinary teams should stop considering this as an error that must be kept quiet, since it is really an area of opportunity to implement additional weight loss strategies that will keep the patient motivated and in better health, instead of waiting for them to regain weight.

Age is a factor frequently associated with surgery success. Younger patients have been reported to have a higher frequency of successful surgeries [7, 8, 12, 14] but we found no correlation between the age and %EBWL 12 months after surgery (R2 = − 0.14, p 0.09). Age alone may not be the sole predictor, considering that with increasing age, more comorbidities and limitations for an intensive diet and exercise are present, [15, 16], while a lower basal energetic expenditure and lipolytic activities are more common [17]. With this information we could also suggest that the goals for the surgery may not be the same in all cases. Older patients may not have very long life expectancies and the goal for their surgery may only be to make their comorbidities easier to manage for their families and primary care physicians, while younger patients also need to be reintroduced quickly to the working force and to reduce the burden of their diseases for many decades more, especially considering that the working status may also influence the eating habits and unemployed or retired patients seem to be more likely to have failed surgeries and weight regain [18, 19]. Our results show also that the %EBWL in the patients with a successful surgery fails to take the patients to a normal BMI. Both groups seem to stay in the range of obesity after 5 years of surgery, the unsuccessful group reaches on average the Grade II of obesity (30–35 kg/m^2^) while the successful group has a nadir in the overweight range but return to Grade I obesity after 5 years. While these numbers may be very significant in terms of controlling their comorbidities, improving quality of life, and life expectancy, they are clearly insufficient to take the patients to a “normal” weight, or even to similar proportions of excess weight compared to the general population. These results require long term evaluations or large cohorts to determine if health improvements persist overtime with surgery alone.

The differences that we found in this study regarding comorbidities between the successful and non-successful groups have been reported by other authors, [14] but the findings are not consistent in all cases [9, 20, 21]. It is noteworthy that these associations are not significant after they were adjusted by age. Diabetes and mental health problems, which are frequently related to difficulties to lose weight after surgery, [7, 9, 20–22] were not a significant differentiating factor in this study.

Regarding surgery types, OAGB was more frequently associated with higher probabilities of reaching a %EBWL > 50%, because it creates a longer malabsorptive loop and is associated with bigger changes in appetite-regulating hormones such as GLP-1 and ghrelin [23, 24]. Weight loss success was followed by RYGB and finally by the gastric sleeve. So far we haven’t found any other publication including these 3 surgeries. It is important to mention that the retrospective nature of this study hinders a homogeneous distribution of confusing variables among each group corresponding to each type of bariatric surgery. By dividing the population into groups according to the type of surgery performed and comparing these groups, we found that in the gastric sleeve group there was a higher proportion of hypertension and hypothyroidism, compared with RYGB and OAGB (data not shown in results); factors that could be associated with a lower success rate in this type of bariatric surgery.

The influence of gender in weight loss has also been a matter of debate [7, 9, 10, 20], in our country, women tend to be more frequently obese than men (ENSANUT 2016) [1] but also they are more likely to be housewives or to have part-time jobs that do not require important physical activities, which may be a distinctive factor in our population. Also, retired patients are more likely to stay at home with their extended family and they are not usually involved in physical activities.

Adherence to diet and exercise is difficult to evaluate [21, 22, 25, 26]. By using self-reports, we were not able to find differences in these aspects between successful and non-successful surgeries. However, we must consider the limitations of self-reports, in particular the 24-h dietary recalls (24HRs) used in this study; they suffer primarily from limitations associated with memory and difficulty in estimating quantities [27]. Participants often omit some foods and beverages consumed and underreporting is more likely to occur than overreporting [27]. And also is the “social desirability bias”, where the patient’s desire to present himself positively to the evaluator (doctor, nutritionist) can lead him to underreporting [27], which can be more marked in those patients who are not losing weight quickly. There is evidence of an underestimation in energy intake between 6 and 25% when comparing 24HRs against much more accurate methods like DLW (doubly leveled water) [28]. Therefore, these results must be taken with reserve and some other strategies must be implemented to evaluate these variables.

Interestingly, 11 (42%) of the 26 patients in the non-successful group, gained weight during follow-up. This group of patients had a higher prevalence of impaired glucose metabolism and a lower %EWL in the first year after surgery, than the patients that reached > 50% EWL in the second year after surgery. It seems that a poor weight loss during the first year after surgery predicts a failure to lose weight even in subsequent years. The authors consider that the patients that stop losing weight right before reaching their goal may require special follow up or additional therapeutic interventions. The cost of care is also important to consider and requires formal investigation in each center, especially in developing countries, where access to care may be difficult due to the large number of patients looking to be evaluated, contrasted with the limited resources available. In our institution there is currently no access to pharmacological therapies for weight loss and the evaluation by mental health specialists is limited by the large number of patients that require their intervention for conditions different to severe obesity. The authors consider that these limitations will need to be reassessed soon in order to have better outcomes in the long term in our institution, but on the other hand, at this point, our study shows a true reflection of the effects of the surgery, without differential effects of other drugs and intensive therapies that may be a common occurrence in other centers. We also consider that our patients are very homogeneous in their characteristics, with most of them having several predictors for failure according to published literature. It is known that homogeneous groups show little or no differences among them and may not be comparable to other groups were most patients are classified as having grade II or III obesity. These distributions, as well as the low proportion of patients with long term follow up limit our ability to make more general conclusions. More time and patients are needed to evaluate the long term results in our group.

The main limitations of this study are its retrospective nature, with the memory biases that this implies and the lack of a standardized method for the evaluation of nutritional and exercise patterns that patients had after surgery.

## Conclusions

Almost 20% of the patients that undergo bariatric surgery will not lose more than 50% of the EBW during the first 12 months. Some of these patients will probably also fail to reach that goal in the subsequent years. The factors influencing that result are still controversial but may be population-specific and worth evaluating in order to establish the best diagnostic, therapeutic and follow-up strategy for every particular center.

## Data Availability

The datasets generated during and/or analyzed during the current study are available from the corresponding author on reasonable request.

## Abbreviations

BMI: Body mass index
EBW: Excess body weight
OAGB: One anastomosis gastric bypass
RYGB: Y-Roux Gastric Bypass
